# A Practical Treatise on the Diseases Peculiar to Women

**Published:** 1847-01

**Authors:** 


Art. III.—*A Practical Treatise on the Diseases peculiar to Women.* By Samuel Ashwell, m.d., &c. Second Edition.—*London,* 1846. 8vo, pp. 737.

In our Journal for April, 1845 (vol. XIX, p. 345), we gave so favorable an opinion of Dr. Ashwell's 'Treatise on the Diseases of Women,' that the speedy appearance of a second edition was far from surprising us. On the contrary, we looked upon this as a matter of course, and accordingly laid the new volume on our table with great satisfaction. Happening, however, to place the old and new copies together, and to look at the last leaf of both, we were struck with the oddness of the identity of the two numerations, and the linear coincidence of the two final pages—each was page 737, and each page 737 contained eleven lines! This roused our suspicions—roused our well-known editorial keenness of scent after pseudo-second editions—and we then proceeded to compare the old and new volumes more carefully. Advancing from the end of the books towards the beginning, we soon had demonstrative evidence, in identity of type, spacing, capitals, italics, catch-words, literal errors, &c., &c., that the two portions we were examining were precisely the same! And as these evidences of identity continued till we had got over more than two thirds of the book, we naturally concluded that we had here a most flagrant instance of that trick of publishers, which we had already, more than once, exposed,—the palming on the public an old book with a new title-page, as a new edition, in a word, a pseudo-second-edition. And yet our conclusion was not altogether correct, as we at length arrived at a point, namely page 208, where the evidences of identity ceased, and marks of obvious difference presented themselves. And so it turns out, that this so-called second edition of Dr. Ashwell's treatise is only a second edition as far as page 208, all the remaining 529 pages being the identical pages, words, letters, of the edition printed and published and sold to us and to our readers in 1844. Now, as this new volume bears on its title-page the words SECOND EDITION, and as the author gives no hint in his preface (which is also headed *Preface to the* SECOND EDITION) that it is *not* a *bonâ fide* reprint throughout, we think we have just grounds of complaint against both the author and publisher, for thus tacitly allowing the public to be misled in fancying they are really buying a complete new edition of a book, when they are in fact only buying a new edition of less than one third of a book. How this curious thing comes about, we know not, and we care not; but come about as it may, we denounce it as wrong, and we feel that it is our duty thus publicly to expose it. We hope and believe that Dr. Ashwell is no party—or at least, if a party, an innocent party—in this transaction; but it is his duty to explain its precise character to the profession.

As a necessary result of this Mezentian mode of editing, we find some odd little anomalies in reading the old part of the volume with reference to its ostensible date of publication. For instance, at page 280 the profession are justly declared to be "under great obligations to Dr. Walshe for one of the ablest and most complete essays on cancer ever published;" and a warm hope is expressed "that no long time will elapse ere its accomplished author presents it to the medical world as a *distinct work*" (reference being made to the 'Cyclopædia of Surgery'); the truth being, as our readers well know, that this hoped-for *work* was actually given to the public one full year before this notice of it was professedly penned; Dr. Walshe's preface bearing date November, 1845, and Dr. Ashwell's November, 1846.

